# Euthanasia of Cats by Australian Veterinarians: A Survey of Current Practices

**DOI:** 10.3390/vetsci10100627

**Published:** 2023-10-19

**Authors:** Hedia Chan, Brianne Marlene Pepper, Michael P. Ward, Anne Quain

**Affiliations:** Sydney School of Veterinary Science, University of Sydney, Camperdown, NSW 2006, Australia; hcha9940@uni.sydney.edu.au (H.C.); bpep8965@uni.sydney.edu.au (B.M.P.); michael.ward@sydney.edu.au (M.P.W.)

**Keywords:** euthanasia, veterinarian, companion animal, feline, cat, end-of-life, premedication, sedation, pentobarbitone, animal welfare

## Abstract

**Simple Summary:**

Veterinarians are frequently called upon to euthanise cats. As the way in which euthanasia is performed can impact the welfare of cats, we sought to describe the contemporary feline euthanasia practices of Australian veterinarians. We also sought to determine factors associated with the administration of premedication or sedation prior to euthanasia. In an online survey of Australian veterinarians, 95.1% had euthanised at least one cat in the previous 12 months, of which 79.0% had performed euthanasia of a cat in the context of an emergency. Nearly all veterinarians euthanised cats using a barbiturate (99.8%). Premedication or sedation was administered in 71.0% and 52.4% of non-emergency euthanasia and emergency euthanasia, respectively. The most common agent used for premedication or sedation in non-emergency euthanasia was tiletamine-zolazepam, while the most common agents used in emergency euthanasia were opioids. Female veterinarians, those working in ‘other’ workplaces, and those in metropolitan locations were more likely to administer premedication or sedation prior to euthanasia. This study identified scope for refining euthanasia techniques to maximise the welfare of cats, their owners or guardians, and the veterinary team members caring for them.

**Abstract:**

We sought to document the contemporary feline euthanasia practices among Australian veterinarians and determine the factors associated with the administration of a premedication or sedation prior to euthanasia. Australian veterinarians who had euthanised at least one cat during the previous 12 months were invited to participate in an anonymous online survey. From 615 valid responses, 585 respondents (95.1%) had euthanised at least one cat in the last 12 months, of which 462 respondents (75.1%) had performed an emergency euthanasia. Intravenous (IV) injection (n = 536, 91.6%) of pentobarbitone sodium (n = 584, 99.8%) was the predominant primary method. Premedication or sedation was administered to cats by 415/585 (71.0%) and 242/462 (52.4%) of respondents in non-emergency and emergency euthanasia, respectively. In a multivariable logistic regression analysis, for non-emergency euthanasia, being female and working in a metropolitan area were significant predictors for administering a premedication or sedation (*p* < 0.001 and *p* = 0.037, respectively). For emergency euthanasia, working in an ‘other’ type of practice was a significant predictor for administering a premedication or sedation (*p* < 0.001). Australian veterinarians vary in their approach to feline euthanasia. There is scope for refinement of euthanasia techniques to maximise the welfare of cats, their owners or guardians, and veterinary team members.

## 1. Introduction

The term ‘euthanasia’—derived from the Greek words ‘eu’ (‘good’) and ‘thanatos’ (‘death’)—describes a ‘good death’ [1]. In veterinary medicine, ‘euthanasia’ usually refers to ‘ending the life of an individual animal in a way that minimises or eliminates pain and distress’ [1]. Indications for performing euthanasia or humane killing of cats include alleviating intractable or terminal suffering, preventing further decline in quality of life, or protecting the health and safety of animals and people [1,2,3,4,5]. As the majority of animals registered with a veterinary practice are ultimately euthanised [6,7], the way that animals are euthanised can have a broad impact on animal welfare.

Veterinarians have ethical, professional, and regulatory obligations to alleviate suffering, which can be achieved by performing euthanasia where appropriate [8]. According to the Australasian Veterinary Boards Council Day-One Competencies, a veterinarian must be able to “recognise when euthanasia is appropriate and perform it humanely and safely” [9]. This requirement aligns with the Day-One Competencies of the Competency-Based Veterinary Education network (CBVE), the Royal College of Veterinary Surgeons (RCVS), and the European Association of Establishments for Veterinary Education’s European Coordinating Committee on Veterinary Training (ECCVT) [10,11,12]. Veterinary graduates must be trained to competently perform euthanasia in a way that safeguards the welfare of patients [2,13]. Yet, the teaching of end-of-life decision making and euthanasia was shown to be highly variable between and within Australasian veterinary schools [13]. In general, New Zealand (NZ) veterinarians rated euthanasia training received at veterinary school as below satisfactory, with 29% reporting receiving no training in sedation protocols for euthanasia [14]. This may lead to variability in euthanasia techniques and protocols.

Veterinarians are frequently required to perform euthanasia or humanely kill feline patients in veterinary practice. For example, 91% of cats registered to private veterinary practices in NZ were euthanised by a veterinarian at the end of their life [6]. In Australia, a high proportion (28%) of cats admitted to shelters, pounds, and rescue organisations are euthanised or humanely killed [15], which is frequently performed by a veterinarian employed or contracted to provide services to the organisation. A survey of small-animal veterinarians in Australia (n = 230) [16] found that euthanasia was performed a median of four times per week. Another study found that NZ veterinarians euthanised a mean number of 7.9 cats per month (range 0–60) [14].

According to the AVMA, a good death is achieved through the application of a ‘humane technique’ resulting in a ‘rapid loss of consciousness’ [1]. The guidelines specify that ‘animal handling and the euthanasia technique employed should minimise the distress experienced by the animal prior to loss of consciousness’ [1]. The preferred primary euthanasia method described for small companion animals by the AVMA is the intravenous (IV) injection of barbiturates and barbituric acid derivatives [1]. For feline patients, this typically requires restraint in a manner that facilitates access to peripheral veins, for example, the cephalic or saphenous veins [17].

While acknowledging that the use of sedatives or anaesthetics that alter circulation may slightly delay the onset of the primary euthanasia agent, the AVMA guidelines state that ‘sedation and/or anesthesia may assist in achieving the best conditions for euthanasia’ [1]. Pre-euthanasia sedation or anaesthesia ‘should be provided whenever practicable’ prior to the administration of the primary euthanasia agent where animal owners are present regardless of the temperament of the animal or in shelter animals which are ‘distressed, dangerous or fractious’ [1]. The method of euthanasia, including the drug or drugs chosen, the route of administration, and premedication or sedation use, as well as handling and restraint of the patient, determines whether the euthanasia is indeed humane and causes ‘a good death’ [17,18,19].

In addition to impacting the welfare of animals and the wellbeing of their owners, caretakers, or guardians [20,21], the way in which euthanasia and humane killing are performed can also impact veterinary team members. In one study, an increased frequency of euthanasia was associated with increased occupational stress among veterinarians [16], though contextual factors may be critical in determining whether the experience is ultimately negative or positive. A qualitative study using focus groups and individual interviews of Canadian veterinarians found that participants reported improved personal wellbeing when they perceived that they had facilitated a ‘good death’ [22]. Conversely, a negative euthanasia experience (such as when complications occur or signs of distress are observed) was associated with a reduced sense of wellbeing, reduced job satisfaction, increased emotional strain, and concerns about detrimental impacts on the client [22].

Euthanasia techniques have been refined to minimise potential adverse effects such as agonal gasping, which may be distressing to observers [1,18], and safeguarding the welfare of patients and those present, including clients and veterinary team members [18]. In particular, the use of pre-euthanasia premedication, sedation, or anaesthesia is considered best practice [1,18,23]. The presence of the owner during euthanasia is increasingly accepted and even encouraged [18,21], recognising the bond between humans and animals.

Compared with dogs in general, feline patients tend to become stressed more easily when in a veterinary setting [24,25,26,27]. Factors that induce stress include being captured and placed in a carrier for transportation, transportation away from a familiar environment into an unfamiliar environment, exposure to novel visual, auditory, and olfactory stimuli, interaction with unfamiliar people, interaction with unfamiliar animals and species (especially in the waiting room), and separation from owners [27,28,29]. Behavioural changes such as vocalisation and aggression were observed by owners pre- and post-veterinary visits [30,31]. These behaviours, described as ‘feline resistance’ in one report [30], caused some owners to delay subsequent veterinary visits in order to avoid causing further distress in their cats. A survey of cat owners (n = 277) found that 95% said that witnessing stress in their cat during a veterinary visit had had a negative effect on them, and almost one third reported that witnessing stress in their cat had put them off visiting the vet [27]. Additionally, the circumstances in which euthanasia is indicated, for example, acute or chronic health conditions, may contribute to feline stress. For example, cats may be suffering from acute or chronic pain, discomfort or feelings of illness such as nausea, breathlessness, and malaise. The euthanasia procedure itself may be associated with stressful, uncomfortable, or painful procedures including physical restraint for intravenous catheterisation or injection and possible extravasation of drugs. Regarding euthanasia, owner, caretaker, or guardian concerns about feline stress may lead to delayed presentation for euthanasia. Initiatives to address feline fear, anxiety, and distress associated with veterinary visits include Cat Friendly Clinic Accreditation programs [32,33] and training for veterinary team members regarding low-stress handling and ‘Fear-Free’ techniques [34,35]. Interventions such as pre-visit pharmaceuticals, premedication, sedation, and even general anaesthesia are increasingly recommended prior to veterinary visits in general to minimise fear, anxiety, and distress in feline patients [28].

There is currently no published literature documenting the feline euthanasia practices of veterinarians in Australia. This study aimed to document the contemporary practices of Australian veterinarians performing non-emergency and emergency euthanasia in feline patients, including the use of premedication or sedation.

## 2. Materials and Methods

### 2.1. Survey Instrument

This cross-sectional study recruited veterinarians registered in Australia to complete an anonymous online survey regarding the techniques utilised to euthanise companion cats and dogs. The findings related to dogs are the subject of a previous paper [36]. This article focuses on the subset of data pertaining to feline patients.

The feline component of the survey consisted of two sections: (1) questions regarding euthanasia techniques utilised for both non-emergency and emergency feline presentations and (2) demographic questions (see Supplementary Table S1). We defined “metropolitan” as major capital cities, “regional” as towns, small cities, and areas that lie beyond the major capital cities, “rural” as open country and settlements of fewer than 2500 residents, and “remote” as places that are considerably out of the way and lacking in major infrastructure.

Survey questions asked about premedication or sedation use, drug of choice and route of administration for the respondent’s most recent euthanasia, setting of the euthanasia consult, the respondent’s workplace, and years since graduation. Participants could select responses from drop-down menus. Where the preferred option was not listed, participants could select ‘Other’ and respond with free text. The final question in section (1) was a free-text response question, ‘Is there anything else you wish to add about your approach in euthanising a cat?’, which was designed as a safety net. The survey was piloted with a veterinary pharmacologist and three companion animal veterinarians who graduated in three different decades (1995, 2005, and 2016). Feedback that clarified questions and was deemed likely to improve data collection was incorporated into the final version of the survey. The survey was designed and managed using Research Electronic Data Capture (REDCap), a secure encrypted server-based application hosted by the University of Sydney.

### 2.2. Recruitment, Consent, and Ethics Approval

Participants were required to be over 18 years old, registered to practice within Australian states and territories, and must have performed at least one euthanasia in the previous 12 months. The link to the survey was distributed through the New South Wales Veterinary Practitioners Board (NSWVPB), Australian Veterinary Association (AVA), Centre for Veterinary Education (CVE) via online newsletters, and through the Australian Veterinary Network (AVN) private Facebook page.

The survey was available online from February to June 2022. Participation was voluntary. Incentives were not offered. Participants could withdraw from the survey prior to submission. Upon completing the survey, an additional link was displayed to a separate survey, in which respondents could provide their email address to receive a summary of the results. Helpline contacts were displayed at the end of the survey for respondents who felt any distress upon recalling their recent euthanasia experiences.

This study was approved by the University of Sydney Human Research Ethics Committee (2021/964).

### 2.3. Data Cleaning

Survey data were downloaded from REDCap into Microsoft^®^ Excel^®^. Only responses from those who had clicked the “submit” button, indicating consent to participate, were analysed.

Where respondents had selected ‘Other’ from the answer drop-down menu and stated an answer already provided in the pre-existing options, responses were reclassified into the pre-existing category. Answers were retained as ‘Other’ if they could not be re-categorised into existing options. The variable “years since graduation” was calculated by subtracting the year of graduation from 2022. The dataset was checked for valid answers prior to importation into IBM SPSS^®^ Statistics Version 28 (release 28.0.0.0).

### 2.4. Descriptive Analysis

Descriptive analyses were performed for demographic data and categorical variables related to euthanasia practices in both non-emergency and emergency situations. The distribution of the continuous variable “years since graduation” was described using summary statistics (median and interquartile range).

### 2.5. Outcome and Explanatory Variables

The outcome of interest was whether a veterinarian had administered a premedication or sedation prior to euthanasing a cat (yes or no).

Four explanatory variables (gender, type of workplace, location, and years since graduation) were considered for regression analysis. Of these, all were categorical with the exception of “years since graduation” (continuous). To facilitate the statistical analysis, some categories were removed or recoded into new categories. The gender category “other” was removed due to a low number of responses. Similarly, for the variable of primary workplace, “animal shelter practice/charity/NGO”, “research laboratory”, and “veterinary teaching hospital” were recoded into “other”. For the variable of location, “rural” and “remote” were recoded into “rural and remote”.

### 2.6. Univariable Analysis

Univariable binary logistic regression analyses were performed to assess the association between the explanatory variables and the outcome. The assumption of linearity for the continuous variable “years since graduation” was checked by categorising the variable based on quartiles, fitting a univariable model, and plotting the resulting odds ratios against the midpoints of the quartile-based groups. Results were reported as odds ratios (OR) and 95% confidence intervals (CI). Estimates were considered statistically significant if the *p*-value was <0.05.

### 2.7. Multivariable Analysis

A multivariable binary logistic regression model was built using a backward elimination approach. The variables were considered statistically significant in the model if the *p*-value was <0.05, and results were reported as odds ratios (OR) and 95% confidence intervals (CI). Potential confounding by “years since graduation” was assessed by calculating the percent change in the regression parameters of variables in the final model when the potential confounder was added to the final model. An estimated change of >20% was considered to indicate substantial confounding, warranting inclusion of the variable in the final model irrespective of its *p*-value. Model value was assessed by comparing the fitted model to the intercept-only model.

### 2.8. Coding of Free-Text Responses

Free-text responses were analysed according to inductive codebook thematic analysis (TA) [37,38]. Briefly, free-text responses were repeatedly read by three authors (HC, BMP, and AQ) to ensure data familiarity. A codebook was developed based on a review of these responses and literature on companion animal euthanasia. Responses were transferred into an MS Word document and updated onto NVivo (Release 1.7.1 (1534) QSR international) to facilitate coding. Comments from a single respondent could be coded multiple times.

Codes were reviewed for internal coherence and distinctiveness from other codes. Where extracts did not fit a code, they were recoded. A table was constructed to depict the codes, frequencies, and examples of coded extracts to indicate the prominence of codes relative to one another. While not typical of a TA approach [39], this approach has been used in previous veterinary studies involving large numbers of free-text responses comprising a large breadth but shallow depth of data [40].

## 3. Results

### 3.1. Demographic Data

There were 615 complete responses pertaining to feline patients, of which 585 respondents (95.1%) had euthanised a cat in the previous 12 months. Therefore, 585 surveys were included in the analysis.

**Figure 1:**
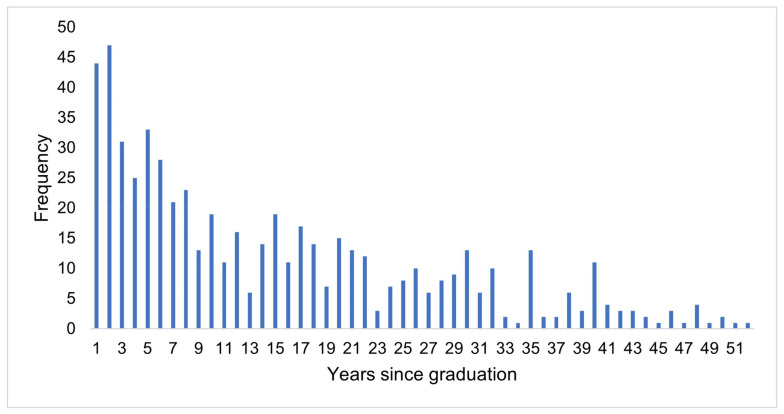
Years since graduation of Australian veterinarians who euthanised a cat in the previous 12 months (n = 585).

The distribution of categorical demographic variables is described in Table 1. Briefly, most respondents were female (n = 485, 82.9%) veterinarians working in private companion animal practice (n = 430, 73.5%). Respondents who chose the ‘other’ job category reported working in a mobile or house-call practice (n = 6), emergency or critical care centre (n = 5), combined emergency and critical care centre and referral hospital (n = 3), corporate companion animal practice (n = 3), combined companion animal and shelter practice (n = 2), combined mixed practice and shelter (n = 1), in-home palliative care and euthanasia service (n = 1), private feline-only practice (n = 1), private equine practice (treating a small number of companion animals) (n = 1), a referral hospital (n = 1), or private small animal only without equine patients (n = 1). Almost half of the respondents worked in a metropolitan (n = 282, 48.2%) or regional location (n = 257, 43.9%), with a minority of respondents working from a rural or remote area (n = 46, 7.9%). The distribution of years of experience since graduation ranged from 1 to 55 years (see Figure 1), with a median of 11 years and an interquartile range of 4 to 22.5 years.

### 3.2. Euthanasia Methods Used by Australian Veterinarians

The distribution of categorical variables describing the techniques used in both non-emergency and emergency euthanasia of cats is described in Table 2 (see Supplementary Tables S2 and S3 for complete data including “other” categories).

Most veterinarians administered a premedication or sedation prior to non-emergency (n = 415/585, 71.0%) and emergency euthanasia (242/462, 52.4%). The primary reason for administering premedication or sedation was to reduce stress to the patient (388/415, 93.3% non-emergency, 218/242, 90.1% emergency) or the owner (327/415, 78.6% non-emergency, 163/242, 66.5% emergency) or as a means of chemical restraint (242/415, 58.3% non-emergency, 139/242, 57.4% emergency).

The most common drugs used for premedication or sedation prior to non-emergency euthanasia were tiletamine-zolazepam (n = 205/415, 49.4%), acepromazine (120/415, 28.9%), and opioids (118/415, 28.4%). The most common drugs used for premedication or sedation prior to emergency euthanasia were opioids (107/242, 44.0%), tiletamine-zolazepam (87/242, 35.9%), and alfaxalone (45/242, 18.6%). In both non-emergency and emergency euthanasia, premedication or sedation was most commonly administered via the intramuscular route (161/415, 38.8% and 107/242, 44.2%, respectively). The next most common route was subcutaneous injection in non-emergency euthanasia (148/415, 35.7%) and intravenous injection in emergency euthanasia (92/242, 38.0%).

Almost all respondents administered pentobarbitone sodium as their primary method of euthanasia (n = 584/585, 99.8%), with most using the intravenous route (536/585, 91.6%). The next most common route of administration was “other” (26/585, 4.4%), of which 23 respondents reported administering the euthanasia drug intra-renally.

The majority of non-emergency euthanasia was performed at the veterinary clinic (n = 531/585, 90.8%), with just under 1 in 10 (49/585, 8.4%) performed as a house call. The owner was present in most cases (513/585, 87.7%). Most respondents reported scheduling 30 min for a non-emergency euthanasia appointment (345/585, 59.0%). The shortest duration reported was 10 min (22/585, 3.8%), while some clinics allocated unlimited time (15/585, 2.6%). In most cases, the veterinarian was assisted (413/585, 70.6%), with veterinary nurses assisting in almost all cases where assistance was provided (405/413, 97.8).

The most common adjunctive measures were ensuring that euthanasia was performed away from other animals (n = 507/585, 86.7%), providing soft bedding for the patient (429/585, 73.4%), and scheduling a longer appointment time (355/585, 60.7%).

Where euthanasia was scheduled, less than 10% of veterinarians dispensed pre-visit pharmaceuticals (n = 56/585, 9.6%); however, of these, most (51/56, 91.1%) dispensed gabapentin.

### 3.3. Factors Associated with the Use of a Premedication or Sedation in a Non-Emergency and Emergency Euthanasia

In the univariable analysis for non-emergency euthanasia, ‘Gender’, ‘Workplace’, and ‘Location’ were significantly associated with use of a premedication or sedation (Table 3). Female veterinarians were 2.5 times more likely than males to use a premedication or sedation (95% CI: 1.6–3.9; *p* < 0.001). Respondents working in ‘Other’ (animal shelter practice/charity/NGO, research laboratory, veterinary teaching hospital) were 2.6 times more likely (95% CI: 1.1–5–9; *p* = 0.006), and respondents from private mixed practices were 1.7 times less likely (95% CI: 0.4–1.0, *p* = 0.006) than respondents who worked in private companion animal practices to use a premedication or sedation. In terms of work location, veterinarians located in metropolitan areas were 2.4 times more likely (95% CI: 1.2–4.5; *p* = 0.004) to administer a premedication or sedation than respondents located in rural and remote areas. ‘Years since graduation’ was not significantly associated with the use of premedication or sedation in a non-emergency cat euthanasia (*p* = 0.702).

In the multivariable analysis, ‘Gender’ (*p* < 0.001) and ‘Location’ (*p* = 0.037) were significant predictors for using a premedication or sedation in a non-emergency cat euthanasia (Table 4). When adjusted for location, females were 2.6 times more likely to administer a premedication or sedation than males (95% CI: 1.6–4.0; *p* < 0.001). When adjusted for gender, respondents from metropolitan areas were 2.2 times more likely (95% CI: 1.0–4.5; *p* = 0.037) to utilise premedication or sedation than rural and remote respondents. There was a good model fit in the multivariable analysis (chi-squared statistic = 33.4, df = 5, *p* < 0.001). Little-to-no evidence of confounding by the years since graduation was observed in this model. There was only a 10.8% change in the regression estimate for ‘Gender’, and 3.0% and 4.4% for ‘Location’ (regional and metropolitan, respectively) in the final multivariable model.

In the univariable analysis for emergency euthanasia (Table 5), ‘Workplace’ and ‘Location’ were significantly associated with premedication or sedation use. Respondents working in ‘Other’ (animal shelter practice/charity/NGO, research laboratory, veterinary teaching hospital) were 4.6 times more likely (95% CI: 1.9–11.2; *p* < 0.001) to give a premedication or sedation to feline patients when compared to respondents working in private companion animal practice. Respondents working in metropolitan areas were 2.9 times more likely (95% CI: 1.4–6.0; *p* = 0.009) to administer a premedication or sedation compared to those from rural and remote areas. ‘Years since graduation’ was not significantly associated with the use of premedication or sedation in an emergency cat euthanasia (*p* = 0.958).

In the multivariable analysis for emergency euthanasia, respondents working in ‘Other’ (animal shelter practice/charity/NGO, research laboratory, veterinary teaching hospital) were 4.6 times more likely (95% CI: 1.8–11.3) to give a premedication or sedation to feline patients when compared to respondents working in private companion animal practice (*p* < 0.001). ‘Location’ was not significantly associated with the use of premedication or sedation in an emergency cat euthanasia (*p* = 0.24).

### 3.4. Free-Text Responses

In total, 140 respondents provided free-text responses, comprising 2829 words. Coding frequencies and examples are provided in Table 6. The most frequent codes were “Premedication, sedation and/or analgesia” and “Use of intravenous catheters”. Opinions, practices, and experiences differed widely between respondents.

## 4. Discussion

This is the first study documenting the feline euthanasia practices of Australian veterinarians. Premedication or sedation was administered by the majority of non-emergency (71.0%) and emergency (52.4%) respondents prior to non-emergency and emergency euthanasias, respectively.

The use of premedication or sedation prior to euthanasia in cats was more frequent than in dogs (68% for non-emergency euthanasia and 47% for emergency euthanasia) [36]. This was also the case in a survey of NZ veterinarians (n = 361), where 47% of veterinarians reported that they always used sedation prior to feline euthanasia (33% in dogs), while just 15% said they would never use sedation for feline euthanasia (20% in dogs) [14]. In general, cats are smaller than dogs and have smaller veins, which may increase the difficulty in achieving venous access. In Canadian animal shelters (n = 67), veterinarians administered pre-euthanasia premedication to cats in 84% of establishments, while trained non-veterinarian staff always administered premedication [41]. This may reflect the skill required to achieve venous access in feline patients. Hypotension, vasoconstriction, and trauma to the site can increase the difficulty of establishing venous access [42]. Veterinary clinical settings, handling, and restraint can trigger fear-associated behaviour in cats, including hissing, swiping, scratching, or biting [43], which impact the safety of those present. The use of premedication or sedation may reduce fear and escalation of these behaviours, minimising the need for restraint and facilitating venous access.

In our study, there were fewer uses of premedication or sedation in emergency euthanasia than non-emergency euthanasia. This aligns with a survey of NZ veterinarians, where 26% of respondents reported that they would be less likely to use premedication or sedation in emergency cases [14]. It is likely in many cases that analgesia or sedation had already been administered as part of the emergency management protocol; therefore, further premedication or sedation was not given prior to euthanasia. Alternatively, cats presented for emergency consultations may be compromised or moribund, such that veterinarians may have been concerned that the administration of such medications may precipitate decompensation or even death.

The key indications for using a premedication or sedation were to reduce stress to the cat, reduce stress to the cat owner, and as a means of chemical restraint in both non-emergency and emergency euthanasia. For example, patients are more relaxed after premedication or sedation, so that the need for physical restraint is reduced [1]. Complications are also less likely to occur, such as agonal gasping, pain, and inadvertent extravasation [44]. A more relaxed patient, and lack of complications, gives the animal, the owner and veterinary team members a better and less stressful experience throughout.

The most common drugs administered prior to non-emergency euthanasia were tiletamine-zolazepam followed by acepromazine and opioids. This differed from emergency euthanasia, where the most common drugs administered were opioids followed by tiletamine-zolazepam and alfaxalone. Tiletamine-zolazepam was the most common premedication or sedation used in cats in NZ (reported by 36% of veterinarians) followed by ‘acepromazine, tiletamine and zolazepam’ (9%), ‘medetomidine and butorphanol’ (8%), ‘ketamine, medetomidine and butorphanol’ (8%), and medetomidine (3%) [14].

Tiletamine is a dissociative anaesthetic, which, when combined with the a benzodiazepine such as zolazepam, provides mild-to-moderate analgesia, muscle relaxation, and chemical restraint in cats [45]. It can be administered IM, SC, and IV and may be effective if delivered intranasally or via buccal administration [46]. Tiletamine increases cardiac output and blood pressure [47], which may improve access to peripheral veins.

The most frequently used drug for premedication or sedation in emergency euthanasia, and the third most commonly used in non-emergency euthanasia, was opioids. It is likely that their more frequent use in emergency euthanasia reflected the patient’s condition, including the presence and severity of pain. In addition to being potent analgesics, opioids may be combined with tranquilisers such as acepromazine to produce synergistic sedative effects (neuroleptanalgesia) [48]. Acepromazine was the second most common drug administered in non-emergency euthanasia and the fourth most common in emergency euthanasia. Used alone, it does not provide analgesia, and there are concerns that it does not result in anxiolysis. It may also cause vasodilation and hypotension, which can increase difficulty in achieving peripheral venous access. However, when combined with opioids, acepromazine enhances sedation and prolongs the opioid analgesic effect [49].

The third most commonly used drug for premedication or sedation in emergency euthanasia was alfaxalone, a synthetic neuroactive steroid that enhances the inhibitory neurotransmitter GABA A complex, causing anaesthesia and muscle relaxation [50]. It is commonly administered intravenously to rapidly induce anaesthesia, but it can also be administered via the IM route [51].

For both non-emergency and emergency euthanasia, most veterinarians administered premedication or sedation via the intramuscular route (38% and 44%, respectively). This finding differs slightly from dogs, where the intravenous route was the predominant route utilised in emergency euthanasia [36]. The reduced frequency of use of the intravenous route in feline patients during emergencies may reflect increased difficulty in establishing venous access in feline patients, particularly those that are critically ill.

While most respondents administered premedication or sedation, a large proportion (29.0% for non-emergency and 47.6% for emergency euthanasia) did not. In a survey of NZ veterinarians regarding euthanasia practices, those who did not use premedication or sedation felt that it was not necessary, that it increased difficulty in achieving venous access, or it was too time consuming [14]. Differences in approaches may be due to the preference of the veterinarian, their previous experiences performing euthanasia (including adverse experiences), drug availability, drug scheduling (including the need to record use of Schedule 8 drugs), onset of action, cost, and variable teaching regarding the administration of premedication or sedation taught in Australasian veterinary schools [13,52]. It has been previously reported that Australasian veterinary students were taught euthanasia by intravenous barbiturate overdose, with or without premedication or sedation [13]. A low response rate to ‘Taught to administer a premedication prior to euthanasia drugs’ as a rationale to use a premedication or sedation may reflect the variable teaching of euthanasia methods and a lack of euthanasia training in veterinary students among veterinary schools [13,14]. It is possible that increased teaching of euthanasia protocols in veterinary schools may increase the frequency of veterinarians administering premedication or sedation prior to euthanasia. Littlewood and colleagues have argued the need for the explicit assessment of euthanasia competency in Australasian veterinary schools [13]. This is echoed by Cooney and colleagues, who call for increased euthanasia education in veterinary schools in the United States [53].

For non-emergency euthanasia, less than 10% of respondents dispensed pre-visit pharmaceuticals. This may be because the decision to euthanise the feline patient was made in the clinic setting or due to owner reluctance or inability to medicate their cat prior to the final visit. Pre-visit pharmaceuticals may lessen feline protective emotional bias and reduce distress during transportation and veterinary visits [43]. However, a global survey of cat owners found that they reported a range of challenges in medicating cats at home, with 77% responding that their cats attempted to bite or scratch them, and 52% of owners reported that administering medication to their cat(s) had altered their relationship with them [54]. In this study, cat owners rated administering tablets as much more difficult to administer than liquids.

Where veterinarians did dispense pre-visit pharmaceuticals, the most common (dispensed in 91% of cases) was gabapentin. Gabapentin inhibits voltage-gated calcium channels in neural tissues. In a randomised, blinded crossover clinical trial, the administration of 100 mg of gabapentin to cats 90 min before transportation to the vet was associated with a significant reduction in stress-related behaviours, both during transport and examination [55]. In addition, it was reported to decrease feline aggression and increase patient compliance. Interestingly, the owner-reported peak effect of the drug was between 2–3 h post administration, suggesting that increasing the time from administration to transport from 90 min to 2–3 h may improve outcomes. Gabapentin may be administered orally as a capsule, or capsule contents may be mixed into food or reconstituted in water to administer orally.

We previously reported that Australian veterinarians dispensed pre-visit pharmaceuticals in 6.9% of dogs prior to non-emergency euthanasia [36]. While their use was slightly higher in cats, these findings suggest that there is scope to increase the use of pre-visit pharmaceuticals in feline patients prior to euthanasia.

The most common primary method of euthanasia was the intravenous injection (n = 536, 91.6%) of pentobarbitone sodium (n = 584, 99.8%). These findings align with a study conducted by Gates and colleagues, who investigated euthanasia protocols in dogs and cats in NZ, where pentobarbitone was the preferred euthanasia drug for cats from 99.2% of veterinarians. [14]. Pentobarbitone, a sedative-hypnotic drug with a narrow margin of safety, is the most commonly used barbiturate acid derivative for the euthanasia of animals [8]. This is consistent with the recommended method of euthanasia by the AVMA Guidelines for the Euthanasia of Animals [1]. Caffery and colleagues found that over half of Canadian animal shelters used pentobarbitone sodium injections (53%) for feline euthanasia followed by T-61 (a mixture of embutramide, mebezonium iodide, and tetracaine hydrochloride) (35%) [41]. T-61 is not available in Australia. Physical methods of euthanasia are rarely used in small companion animals due to welfare, aesthetic, and regulatory concerns, such as operator skill and firearms licensing [1,41,56].

The intravenous route was the most commonly used route of drug administration in cats in private practice veterinary settings [8]. However, for the reasons discussed previously, it may be difficult to establish intravenous access and maintain this access in feline patients due to their physical condition as well as their behaviour.

The AVMA states that intraorgan injections, such as intracardiac, intrahepatic, and intrarenal injections, are only acceptable in an unconscious or fully anaesthetised animal [1]. Concerningly, some respondents reported performing intraorgan injections in conscious patients, which may cause pain and distress to cats. This suggests a need to ensure that Australian veterinarians are aware that these routes should only be used to administer euthanasia drugs in anaethetised or unconscious patients.

Intrarenal and intrahepatic routes are easier to administer but may prolong time to death (about 30 to 60 s and 2 min, respectively) when compared to an IV administration (within 30 s) [57]. In a retrospective study of 131 client-owned cats where pentobarbitone sodium was administered intrarenally [58], 79% of the feline patients had a time to cardiopulmonary arrest (TCPA) of less than a minute, and the remaining cats had a TCPA between 1.5 to 8 min (21%). Therefore, intrarenal administration was considered a good alternative for the IV injection of pentobarbitone sodium in anaesthetised cats. The advantages of this method include allowing owners to hold animals and avoiding the need to clip fur over peripheral veins.

Most veterinarians euthanised cats in a veterinary hospital (90.8% of non-emergency euthanasia compared to 94.6% of emergency euthanasia). This finding was expected, as veterinarians are less equipped to manage emergency cases in non-clinical settings. The higher frequency of cats euthanised off-site in non-emergencies may reflect the growth of in-home euthanasia (IHE) services in Australia. Given that the majority of feline euthanasia occurs in veterinary clinical settings, it is important to ensure that a suitable, feline-friendly environment is available [59]. If an appropriate space is not available within the veterinary clinical setting, a house call or referral to an IHE provider may provide a less stressful experience for both the patient and the client.

Owners were present in the majority of non-emergency (87.7%) and emergency (81.4%) euthanasias, findings that were similar to dogs. While historically, owner presence was discouraged, it is now considered best practice to keep the cat and owner together where possible during the euthanasia consultation [18]. This recommendation is supported by a randomised crossover trial, which reported that separation from their owner, coupled with being removed to the common treatment area, significantly increased heart rate and behaviours associated with fear, anxiety, and stress in cats [31]. The authors recommended that wherever possible, physical examinations and procedures on cats should be performed with the owners present and away from dogs [31].

Most of the time, veterinary nurses assisted veterinarians in performing both non-emergency and emergency euthanasia. This highlights the need for training and support for veterinary nurses to assist in euthanasia, including appropriate cat handling. Approximately one third of veterinarians were not assisted in performing their most recent feline euthanasia. It may be that assistance was not required due to the behaviour or condition of the cat (including whether the patient was sedated or anaesthetised). However, it is possible that assistance was not available (for example, if the euthanasia occurred after hours or in the context of a staff shortage). These possibilities highlight the need for euthanasia protocols that can be employed without an assistant. For example, the use of premedication or sedation may eliminate the need for assistance by providing chemical restraint. Very few veterinarians were assisted by clients. Clients may have assisted because there was no alternative assistant available, or they may have assisted due to a wish to take a more active role in the euthanasia process. Veterinarians involving clients in euthanasia need to consider both patient and client safety.

Respondents frequently used adjunctive or non-pharmacological measures to improve the euthanasia experience for both feline patients and clients. The most commonly reported were soft bedding, performing euthanasia away from other animals, and a longer appointment time. This was similar to the findings we reported for dogs [36], although treats were more commonly provided to canine patients (60%) compared to feline patients (13.8%).

Most respondents scheduled 30 min for feline euthanasia, though time scheduled ranged from 10 min to unlimited. A study on clients from 14 veterinary clinics in Canada [21] reported that one third of the respondents wanted time to farewell their pet. A survey of companion animal owners by Matte and colleagues [20] reported that clients ranked being able to spend time alone with their pets in veterinary settings second only to receiving reassurance as the most supportive thing veterinarians could do in relation to euthanasia. Yet, only 72% clients felt they received alone time with their pets in the euthanasia consultation [20]. Cooney and Kipperman propose scheduling >45–60 min for euthanasia to allow for a discussion of the prognosis, shared decision-making, and informed consent [44]. They contend that clients may become frustrated and upset if they perceive the euthanasia appointment to be hurried. They state that these consultations “have evolved to become pseudo-funerals: unique, emotional medical procedures in full view of owners unlike anything else undertaken in veterinary medicine” [44]. These evolving expectations around euthanasia may be challenging for veterinarians to accommodate, particularly in the face of high caseloads and staff shortages. For non-emergency euthanasias, some conversations, including information about the euthanasia procedure, could take place by telephone, or alternatively they may involve other veterinary team members such as veterinary nurses. These discussions may help veterinary team members in creating individualised euthanasia plans for feline patients, ensuring that all team members are aware of both patient and client needs and preferences [60]. Scheduling of non-emergency feline euthanasia during less busy periods may benefit feline patients, clients, and even veterinary team members in providing a suitable environment with minimal disturbances and potential stressors and reduced time pressure.

The free-text responses revealed variations in views regarding the aspects of euthanasia, including premedication or sedation and intravenous catheter use. An NZ study found highly divergent views around the placement of intravenous catheters, appropriate methods of patient restraint, and the question of whether owners should be present during companion animal euthanasia [14].

Among the four demographic variables explored, ‘Gender’, ‘Workplace’, and ‘Location’ were significantly associated with the use of a premedication or sedation in a non-emergency euthanasia. Female veterinarians were 2.5 times more likely to administer premedication or sedation than males. In previous studies, female veterinarians tended to estimate pain as more severe and treat animals for pain more frequently than their male counterparts [61,62,63,64]. It has been suggested that female veterinarians have higher levels of empathy [64]. Veterinarians whose primary workplace was a private mixed animal practice were least likely to administer a premedication or sedation, while those working in the “other” category were most likely to do so. This cohort included veterinarians providing house-call services, who may also provide IHE, as well as animal welfare veterinarians and those working in academia. It is possible that these groups are more likely to be aware of current euthanasia guidelines. Our results showed an increasing tendency for premedication or sedation administration as workplace locations shifted from rural and remote areas via regional to metropolitan areas, with metropolitan areas having a significantly higher odds ratio of using premedication or sedation. This could be due to less access to veterinarians in rural and remote areas [65,66] or possibly to an increased proportion of financial constraints in clients. However, further study is required to explore this association.

The only predictor significantly associated with the administration of a premedication or sedation prior to emergency euthanasia was workplace, with veterinarians in the “other” category being 4.6 times more likely to give a premedication or sedation compared to those in private companion animal practice. We anticipate that this is due to similar reasons to those discussed for non-emergency euthanasia.

One unexpected finding was that “years since graduation” was not associated with an increased likelihood of administration of premedication or sedation. We expected that more recent graduates (those with fewer years since graduation) would be more likely to be aware of current guidelines and thus be more likely to administer a premedication or sedation than veterinarians graduating earlier (those with more years since graduation).

The discrepancy between current recommendations to premedicate or sedate animals before euthanasia and clinical practice suggests a need for additional training to improve veterinarians’ confidence and familiarity with premedication or sedation, including appropriate doses and routes of administration, to maximise the benefits and minimise the potential adverse effects. Increasingly, veterinarians have opportunities to engage in euthanasia-focused continuing professional development (CPD). As an example, the Companion Animal Euthanasia Training Academy (CAETA) (https://caetainternational.com/, accessed 30 August 2023) is an example of an organisation that provides training specifically on end-of-life care and euthanasia in companion animals. Within Australia, the Australian Veterinary Palliative Care Advisory Council (AVPCAC) provides intermittent CPD on euthanasia and online resources for veterinary team members (https://www.avpcac.com/, accessed 30 August 2023).

To encourage the use of premedication or sedation, the factors underlying the differences identified could be explored in future studies. For example, if veterinarians working in mixed practice or those in rural and remote locations are less likely to administer a premedication or sedation to feline patients due to impediments such as lack of time or staff shortages, providing additional training without addressing those impediments may not increase the uptake of these practices.

## 5. Limitations

While this study represents a reasonable proportion of veterinarians (n = 585, representing approximately 4.2% of the 13,933 registered veterinarians in 2021 [67]), the results must be generalised with caution. Nonetheless, the demographic of our respondents is consistent with the Australian Veterinary Association’s 2021 Workforce Survey analysis (n = 3749), with respondents to both surveys being predominantly female practitioners working primarily with companion animals [67]. The demographic is also similar to veterinarians in other countries, including the UK, the US, and NZ [68,69,70].

Voluntary surveys are vulnerable to non-response bias, only reflecting the views of those motivated to respond to the survey [71]. Veterinarians who found euthanasia stressful to recall may have been disinclined to participate, potentially biasing the results. Conversely, veterinarians who may have a particular interest in companion animal or feline euthanasia, including those that have undertaken additional training, may have been more motivated to respond.

Retrospective surveys are subject to recall bias [71]. To minimise recall bias, respondents were asked to recall their most recent feline euthanasia cases in the last 12 months. Survey questions regarding premedication or sedation use may be subject to social desirability bias [71]. Respondents may have reported what they believed the investigators thought should have been done rather than report on what they actually did. The use of pre-euthanasia premedication or sedation has been reported to promote patient welfare [1,18,22,23], client wellbeing [18,22,23], and the wellbeing of veterinary team members [22], which may have influenced the responses. Survey anonymity may limit social desirability bias. One disadvantage of anonymity is that we were unable to clarify ambiguous free-text responses.

The survey did not include a definition of a non-emergency and emergency euthanasia. While this was not an issue identified during survey piloting with veterinarians, the respondents may have varied in what they considered to be a non-emergency or emergency euthanasia. This reduced the precision with which we could compare the techniques used in non-emergency and emergency euthanasia.

Due to an error in the branching logic in the REDCap survey, data associated with adjunctive factors (including client presence and assistance) during the emergency euthanasia were not collected from respondents who performed an emergency euthanasia but did not use a premedication or sedation. Therefore, any significant differences between non-emergency and emergency euthanasia regarding these factors could not be explored.

We sought to minimise survey attrition by restricting the number of questions to a minimum. It would be useful to determine the frequency of feline euthanasia per veterinarian in future studies. The mean number of cats euthanised by NZ veterinarians was 7.9 per month (median 5, range 0–60) [14]. The frequency of euthanasia may influence the technique used. Additionally, it would be useful to collect data regarding the dose rates of drugs used for premedication or sedation and their efficacy. It is possible that in cases where premedication or sedation was administered, the dose may not have been appropriate to achieve the desired effect. It would also be helpful to collect information about the rate of administration of pentobarbitone sodium, as this may be associated with adverse effects such as agonal gasping [72]. Furthermore, our survey only focused on reasons for using a premedication or sedation but did not investigate reasons for not using a premedication or sedation. In NZ, while 45% of veterinarians worked in a setting with a feline euthanasia protocol, 96% of those reported they were likely to follow this protocol, suggesting the utility of euthanasia protocols [14]. To develop, standardise, and refine feline euthanasia protocols, it would be useful to collect data on dose rates, rates of administration, and both indications and contraindications for the administration of premedication or sedation prior to euthanasia.

We did not survey veterinarians about their perceptions of the quantity or quality of training in euthanasia techniques undertaken when they were veterinary students or graduates. A study of NZ veterinarians found that most ranked their undergraduate training as unsatisfactory, with 30% receiving no training on sedation protocols for euthanasia [14]. Most learned from their colleagues or employer, with 83% reporting they had changed their euthanasia technique since graduation, yet 74% reported having no formal training in euthanasia techniques after graduation. The most common reported change in euthanasia techniques since graduation was adopting pre-euthanasia premedication or sedation.

This study was conducted during the COVID-19 pandemic (February to June 2022); hence, the euthanasia experiences that the respondents recalled would be within the period from February 2021 to June 2022. It is possible that during this time, to facilitate physical distancing and comply with public health orders, some veterinarians modified their euthanasia practices, performing low- or no-contact euthanasia [73]. For example, premedication or sedation may have been used to facilitate the placement of an intravenous catheter and an extension set so that pentobarbitone could be administered at a distance from the cat being held by the owner. Therefore, our results may have overestimated the use of premedication or sedation used by Australian veterinarians outside of pandemic-associated restrictions.

## 6. Conclusions

Most Australian veterinarians euthanised feline patients with pentobarbitone sodium, while 71.0% administered pre-euthanasia premedication or sedation in non-emergencies, and 52.4% did so in emergencies. Not all the respondents utilised premedication or sedation prior to intraorgan administration of pentobarbitone sodium, as recommended in current guidelines. This demonstrates a need for further training in feline euthanasia techniques to ensure that Australian veterinarians are confident and competent in performing best-practice euthanasia, including premedication and sedation and intraorgan administration of pentobarbitone sodium. Veterinarians need access to appropriate training and continuing professional development around the technical aspects of feline euthanasia. Additionally, there is potential scope to increase the appropriate administration of pre-visit pharmaceuticals.

The development, standardisation, and refinement of feline euthanasia protocols will assist in minimising adverse effects and maximising the benefits associated with both premedication, sedation, and euthanasia.

The refinement of euthanasia practices also requires addressing financial and practical constraints that may limit the use of premedication and sedation.

These findings allow individual veterinarians and veterinary teams to compare their practices around euthanasia, both pharmacological and non-pharmacological, against those of their peers. It will assist in the development, standardisation, and refinement of feline euthanasia protocols.

## Figures and Tables

**Table 1:** Categorical demographic information of Australian veterinarians who euthanised a cat in the previous 12 months (n = 585).

Demographic Parameter	Category	Number	Percentage (%)
Gender	Male	97	16.6
	Female	485	82.9
	Other	3	0.5
Primary workplace	Animal shelter practice/charity/NGO	16	2.7
	Private companion animal practice	430	73.5
	Private mixed practice	103	17.6
	Research laboratory	1	0.2
	Veterinary teaching hospital	10	1.7
	Other (please specify)	25	4.3
Location	Metropolitan	282	48.2
	Regional	257	43.9
	Rural	45	7.7
	Remote	1	0.2

**Table 2:** Descriptive statistics (count and %) relating to the most recent emergency and non-emergency feline euthanasia performed by Australian veterinarians in the previous 12 months (n = 585).

Category		Non-Emergency Euthanasia		Emergency Euthanasia	
		Count/Denominator	Percentage (%)	Count/Denominator	Percentage (%) **
Did you use premedication or sedation?	Yes	415/585	71	242/462	52.4
	No	170/585	29	220/462	47.6
What was the reasoning behind using a premedication?	Chemical restraint	242/415	58.3	139/242	57.4
(Participant could select multiple options)	Clinic protocols	54/415	13	24/242	9.9
	Reduce stress to owner	327/415	78.6	163/242	66.5
	Reduce stress to patient	388/415	93.3	218/242	90.1
	Taught to administer a premedication prior to euthanasia drugs	32/415	7.7	11/242	4.5
	Other (please specify)	26/415	6.3	24/242	9.9
What was the drug used for premedication or sedation prior to the euthanasia you performed most recently?	Acepromazine	120/415	28.9	43/242	17.8
(Participant could select multiple options)	Alfaxalone	61/415	14.7	45/242	18.6
	Inhalation anaesthesia	2/415	0.5	7/242	2.9
	Ketamine	31/415	7.5	21/242	8.7
	Medetomidine/dexmedetomidine	61/415	14.7	35/242	14.4
	Opioids (methadone, buprenorphine, tramadol, butorphanol, pethidine)	118/415	28.4	107/242	44
	Propofol	20/415	4.8	13/242	5.4
	Tiletamine-zolazepam	205/415	49.4	87/242	35.9
	Thiopentone	10/415	2.4	8/242	3.3
	Xylazine	9/415	2.2	5/242	2.1
	Other (please specify)	15/415	3.6	1/242	0.4
What was the route of administration for the premedication that you used?	Inhalation	2/415	0.5	8/242	3.3
(Participant could select multiple options)	Intracardiac injection	1/415	0.2	2/242	0.8
	Intravenous injection	115/415	27.7	92/242	38
	Intramuscular injection	161/415	38.8	107/242	44.2
	Intraperitoneal injection	2/415	0.5	2/242	0.8
	Oral administration	4/415	0.95	0/242	0
	Subcutaneous injection	148/415	35.7	45/242	18.6
	Other (please specify)	0/425	0	2/242	0.8
What was the primary method that you used in your most recent non-emergency euthanasia of a cat?	Pentobarbitone sodium	584/585	99.8	-	-
(Participant could select multiple options)	Anaesthetic inhalation	2/585	0.3	-	-
	Potassium chloride	1/585	0.2	-	-
	Thiopentone	3/585	0.5	-	-
	Other (please specify)	1/585	0.9	-	-
What was the route of administration of your chosen euthanasia drug?	Intravenous injection	536/585	91.6	-	-
(Participant could only select one option)	Inhalation	0/585	0	-	-
	Intracardiac injection	15/585	2.6	-	-
	Intramuscular injection	1/585	0.2	-	-
	Intraperitoneal injection	7/585	1.2	-	-
	Oral administration	0/585	0	-	-
	Subcutaneous injection	0/585	0	-	-
	Other (please specify)	26/585	4.4	-	-
Was the euthanasia a house call or did it happen at the clinic?	House call	49/585	8.4	13/242	5.4
(Participant could only select one option)	At the clinic	531/585	90.8	229/242	94.6
	Other (please specify)	5/585	0.85	0/242	0
Was the owner present during the euthanasia?	Yes	513/585	87.7	197/242	81.4
	No	72/585	12.3	45/242	18.6
How long do you schedule for a routine euthanasia?	10 min	22/585	3.8	-	-
(Participant could only select one option)	20 min	113/585	19.3	-	-
	30 min	345/585	59	-	-
	40 min	28/585	4.8	-	-
	60 min	23/585	3.9	-	-
	Other (please specify)	39/585	6.7	-	-
	Unlimited	15/585	2.6	-	-
Were you assisted during the euthanasia?	Yes	413/585	70.6	168/242 *	69.4
	No	172/585	29.4	74/242 *	30.6
Who assisted?(Participant could only select one option)	Client	3/413	0.7	5/168 *	3
	Veterinary nurse	405/413	97.8	162/168 *	96.4
	Other (please specify)	5/413	1.2	1/168 *	0.6
What adjunctive measures did you take to minimise fear/anxiety/stress in the patient?	Away from other animals	507/585	86.7	206/242 *	85.2
(Participant could select multiple options)	Pheromones	207/585	35.4	78/242 *	32.1
	Dim lighting	128/585	22	48/242 *	19.8
	Longer appointment time	355/585	60.7	116/242 *	47.7
	Soft bedding	429/585	73.3	165/242 *	67.9
	Soft music playing	22/585	3.8	7/242 *	2.9
	Treats	81/585	13.8	24/242 *	9.9
	Cat only consult room	141/585	24.1	44/242 *	18.1
	ISFM accreditation	33/585	5.6	11/242 *	4.9
	Other (please specify)	42/585	7.2	14/242 *	5.8
	None	26/585	4.4	17/242 *	7
Did you dispense medication to the owner for the patient prior to the appointment? (Participant could select multiple options)	Barbiturates	1/56	1.8	-	-
	Clonidine	0/56	0	-	-
	Gabapentin	51/56	91.1	-	-
	Opioids	2/56	3.6	-	-
	Oral acepromazine	1/56		-	-
	Trazodone	0/56	0	-	-
	Other (please specify)	0/56	0	-	-
	None	529/585	90.4	-	-

* Due to an error in branching logic, responses were only selected for those who administered a premedication. Abbreviations: ISFM = International Society for Feline Medicine. ** Column percentage can add to over 100% where participants could select multiple options.

**Table 3:** Descriptive results and univariable logistic regression results for demographic variables associated with the use of premedication in the most recent non-emergency euthanasia of a cat by Australian veterinarians (n = 582).

		Premedication or Sedation			Univariate	
Predictor	Category	Yes (%)	No (%)	Total	OR (95% CI)	*p* Value
Gender	Male	52 (53.6)	45 (46.4)	97	1.0	<0.001
	Female	360 (74.2)	125 (25.8)	485	2.5 (1.6–3.9)	–
Workplace	Private companion animal practice	304 (71.2)	123 (28.8)	427	1.0	0.006
	Private mixed practice	63 (61.2)	40 (38.8)	103	0.6 (0.4–1.0)	
	Other *	45 (86.5)	7 (13.5)	52	2.6 (1.1–5.9)	
Location	Rural and remote	27 (58.7)	19 (41.3)	46	1.0	0.004
	Regional	170 (66.1)	87 (33.9)	257	1.4 (0.7–2.6)	
	Metropolitan	215 (77.1)	64 (22.9)	279	2.4 (1.2–4.5)	
Years since graduation	0–4 years	103 (71.0)	42 (29.0)	145	1.0	0.702
	5–11 years	101 (68.2)	47 (31.8)	148	0.9 (0.5–1.4)	
	12–22 years	107 (74.3)	37 (25.7)	144	1.2 (0.7–2.0)	
	23+ years	101 (69.7)	44 (30.3)	145	0.9 (0.6–1.6)	

* The ‘Workplace’ variable ‘Other’ category included ‘Animal shelter practice/charity/NGO’, ‘Research laboratory’, and ‘Veterinary teaching hospital’.

**Table 4:** Final binary multivariable logistic regression results for demographic variables associated with the use of premedication in the most recent non-emergency euthanasia of a cat by Australian veterinarians (n = 582).

Predictor	Categories	Adjusted OR	95% CI	*p* Value
Gender	Male	1.0		<0.001
	Female	2.6	1.6–4.0	
Location	Rural and remote	1.0		0.037
	Regional	1.3	0.7–2.7	
	Metropolitan	2.2	1.0–4.5	

**Table 5:** Descriptive results and univariable logistic regression results for demographic variables associated with the use of premedication in the most recent emergency euthanasia of a cat by Australian veterinarians (n = 461).

		Premedication or Sedation			Univariate	
Predictor	Category	Yes (%)	No (%)	Total	OR (95% CI)	*p* Value
Gender	Male	34 (42.5)	46 (57.5)	80	1.0	0.055
	Female	207 (54.3)	174 (45.7)	381	1.6 (1.0–2.6)	
Workplace	Private companion animal practice	177 (53.2)	156 (46.8)	333	1.0	<0.001
	Private mixed practice	33 (36.3)	58 (63.7)	91	0.5 (0.3–0.8)	
	Other *	31 (83.8)	6 (16.2)	37	4.6 (1.9–11.2)	
Location	Rural and remote	13 (32.5)	27 (67.5)	40	1.0	0.009
	Regional	112 (50.2)	111 (49.8)	223	2.1 (1.0–4.3)	
	Metropolitan	116 (58.6)	82 (41.4)	198	2.9 (1.4–6.0)	
Years since graduation	0–4 years	54 (50.9)	52 (49.1)	106	1.0	0.958
	5–11 years	68 (51.5)	64 (48.5)	132	1.0 (0.6–1.7)	
	12–22 years	57 (52.3)	52 (47.7)	109	1.1 (0.6–1.8)	
	23+ years	62 (54.4)	52 (45.6)	114	1.1 (0.7–2.0)	

* The ‘Workplace’ variable ‘Other’ category included ‘Animal shelter practice/charity/NGO’, ‘Research laboratory’, and ‘Veterinary teaching hospital’.

**Table 6:** Codes, frequencies, and examples of free-text comments in response to the question “Is there anything you wish to add” regarding euthanasia techniques used by Australian veterinarians on feline patients (n = 140).

Code	Number of Comments Coded	Examples
Premedication, sedation, and/or analgesia	68	*“Often old cats have the worst veins, and they are also arthritic and hate their legs being handled. I think they need to be given sedation/pain relief.”* (8) *“In the ideal world. the cats would be given gabapentin prior to presenting—most times, it doesn’t happen due to the owners making the decision with not giving us much time to prepare—they seem to give more time for their dogs than their cats.”* (37) *“Less sedation for old sick cats.”* (369) *“…never use sedation.”* (470) *“I do not understand why some vets do not use sedation. I used to not due to lack of training and doing what others did. My eyes were opened by my previous boss. Now I would not do a euthanasia without it.”* (607)
Use of intravenous catheters	47	*“Always place an IV catheter. This is done in a separate room before cat is brought back to owner.”* (46) *“I primarily use premedication to allow catheter placement with minimum stress. Most emergency euthanasias already have a catheter placed.”* (151) *“[I] find catheter placement in saphenous vein much better tolerated.”* (335)
Methods of euthanasia (including route of administration)	29	*“I always try to go IV but warn owners that sometimes I have to go into the kidney.”* (21) *“If the cat is untouchable, I would use volatile anaesthesia in a box. If the veins can’t be catheterized, I would do a lethal injection intracardiac.”* (35) *“I often use a direct injection into the kidney (care to not inject into renal pelvis) which I find works well with no obvious pain or distress.”* (376) *“In my area, there are a lot of feral cats. The landowner catches the cat in a live-catch cage and signs appropriate paperwork. I check the cat is genuinely feral and not tame. (If tame I take it to the pound for desexing and rehoming). Euthanasia is instant loss of consciousness with a single 0.22 rifle subsonic (quiet) shot to the base of the brain. I try to minimise stress to the cat by keeping its cage covered and approaching quietly and slowly.”* (696)
Euthanasia of anxious or aggressive animals	21	*“We use high doses of premeds to heavily sedate many unsocialised and feral cats presented to us.”* (262) *“Our clinic performs a lot of feral cat euthanasias. I always administer 0.5 mL xylazine IM prior to handling the cat. 1. For safety—have had too many nurses and vets bitten/scratched. 2. Reduce the stress to the cat when handling and going IC with Lethabarb [pentobarbitone sodium] injection”* (357). *“Sometimes I use Zoletil [tiletamine/zolazepam], particularly if in a hurry. For really aggressive cats I have dispensed gabapentin 100 mg capsule for the client to administer prior to doing the job. For feral cats I have sometimes used a Jabstick [a push-operated pole syringe] to safely administer anaesthetic. If no owner present then I don’t mind using xylazine to sedate the cat, they will always vomit with this protocol but it is a great drug and once anaesthetised, I have no issue with intracardiac pentobarbitone if no owner present (animal must be anaesthetised first).”* (698)
Communicating with clients	15	*“I spend a lot of time prior to the injection making sure all present are in agreement, understand the necessity, humanity and what will happen.”* (20) *“Allow time to spend alone with the cat beforehand (presuming it is not an emergency euthanasia) explaining what the procedure is, and being compassionate.”* (100)
Minimising patient fear, anxiety, or distress	13	*“We always use stress free handling techniques with cats.”* (139) *“Use of minimal restraint, medial saphenous vein.”* (179) *“I don’t normally give a premed but I think it was a good call and the cat fell asleep eating treats and I felt it was a good death.”* (513)
Approaches to euthanasia are unique or dependent on individual patient	11	*“Every single one different”* (276). *“Every case is different and there is no one blanket treatment. I use a lot of medetomidine IM after hours to sedate the patient before I put an IV in. I don’t have access to nurses on call. Moribund cats I go intra-kidney often with the owners present.”* (610). *“Each cat’s character and nature is taken into account when determining the best approach to euthanise them. I always try to make it as stress free and caring as possible.”* (646)
Euthanasia of unowned or stray animals	11	*“With shelter animals I try to treat them as if they are owned.”* (117) *“Our clinic routinely euthanises strays presented by the pound. These animals are in crush cages and are given intraperitoneal/intrarenal/intrahepatic injections of pentobarbitone without any sedation. The cages are covered with towels to provide shelter for them. This procedure is clinic policy.”* (138) *“Shelter euthanasias are very different to client directed euthanasia in my experience.”* (623)
Minimising owner stress	10	*“Placing an IV canula in patient in a room away from owner minimises stress to owner then adding an extension tube so euthanasia solution can be administered at a distance and not between owner and cat at time of administering pentobarbitone.”* (63) *“Owner engagement important, allow owner to stay with cat after heart stopped.”* (699)
Assistance during euthanasia	8	*“…decision to use or not use premed depends on condition/demeanour of cat, availability of assistant (not owner) for restraint if required.”* (14) *“IV catheter placed with vet nurse restraining. If cat fractious/distressed will admin IM or SC sedation before placing catheter.”* (244)
Adverse effects and their management or avoidance	6	*“I buffer my premedication to reduce the sting.”* (16) *“May consider adding a sedation prior to any euthanasia to prevent gasping or reactions during procedures, not happen that often but it is very distressing.”* (565) *“I place the IV catheter out of the consult room in case the cat flinched, reacts to the needle. We also use EMLA cream to reduce feeling.”* (630)
Do not separate animal from owner at any point in the process	5	*“Never take cat away from owner.“* (5) *“Intraperitoneal route is my favoured route for cats. No pain, no restraint required, goes to sleep over a few minutes during which time many owners want to hold and cuddle their pet.”* (82).
Discussing aftercare of the body costs and paying accounts	4	*“All the discussion of aftercare and payment is done prior.”* (20) *“I spend a lot of time before and after the euthanasia talking to owner (and family) about after-care options, grief support, memorial items and supporting their other pets.”* (428)
Time of appointment	4	*“I try not to rush clients. I love home euthanasias but no longer work in a clinic that offers this.”* (462) *“I put everything I can in for the patient and the client in that 30 min.”* (607)
Location of euthanasia	4	*“Would prefer cat only rooms.”* (373) *“I would prefer to perform all euthanasias in the pet’s home rather than a clinical setting.”* (59)
Indications and justifications for euthanasia	3	*“I must convince myself there is nothing I could help the cat to alleviate the suffering and pain, also there is no quality of life.”* (6) *“Advise [that euthanasia is] a just act. Listen to their story.”* (69)
Need to be compassionate in general	3	*“gentle hands & kind words.”* (15) *“do it well, smoothly kindly, with sympathy.”* (705)
Sympathy cards and memorials	2	*“In our clinic we offer gold paw prints and hair samples and send sympathy cards. We send flowers to significant pets (long association with the clinic). I have also recently started to take photos before and after [euthanasia] with the client’s permission and have printed these out and given to the client. I have found this very well received.”* (65)
Minimising stress to veterinary team members including self	1	*“I have to mentally prepare myself for euthanasia every single time.”* (607)

Key: IV = intravenous; IM = intramuscular; IC = intracardiac; SC = subcutaneous.

## Data Availability

The data are unavailable due to ethical restrictions.

## Supplementary Materials

The following supporting information can be downloaded at: https://www.mdpi.com/article/10.3390/vetsci10100627/s1. Table S1: Survey Questions - Euthanasia of cats by Australian veterinarians: a survey of current practices; Table S2: Descriptive statistics of euthanasia practices of the most recent non-emergency euthanasia of a cat by Australian veterinarians (n = 615). Table S3: Descriptive statistics of euthanasia practices of the most recent emergency euthanasia of a cat by Australian veterinarians (n = 585).
