# Supplementary material for: Euthanasia of Cats by Australian Veterinarians: A Survey of Current Practices
Source: Vet Sci. 2023 Oct 19;10(10):627. doi: 10.3390/vetsci10100627 (PMC10610671; doi:10.3390/vetsci10100627)
Supplement: Supplementary file 1 [file vetsci-10-00627-s001.zip › vetsci-2621560-supplementary.pdf]

Supplementary information

Table S1. Survey Questions - Euthanasia of cats by Australian veterinarians: a survey of current practices.

Have you euthanised a cat in the last 12 months?	Yes<br>No
Non-Emergency Euthanasia	
For the most recent, non-emergency euthanasia you performed in a cat, did you use premedication or sedation?	Yes<br>No
What was the reasoning behind using a premedication?	Chemical restraint<br>Clinic's protocols<br>Reduce stress to owner<br>Reduce stress to patient<br>Taught to administer a premedication prior to euthanasia drugs<br>Other (please specify)<br>(Please select all that apply.)
Please specify your answer.	(Free text response)
What was the drug used for premedication or sedation prior to the euthanasia you performed most recently?	Acepromazine<br>Alfaxalone<br>Inhalation anaesthesia<br>Ketamine<br>Medetomidine / Dexmedetomidine<br>Opioids (Methadone, buprenorphine, tramadol, butorphanol, pethidine)<br>Propofol<br>Tiletamine-zolazepam<br>Thiopentone<br>Xylazine<br>Other (please specify)<br>(If you have used a combination of drugs, please select each of these.)
Please specify your answer.	(Free text response)
What was the route of administration for the premedication that you used?	Inhalation<br>Intracardiac injection<br>Intravenous injection<br>Intramuscular injection<br>Intraperitoneal injection<br>Oral administration<br>Subcutaneous injection<br>Other (please specify)<br>(Please select all that apply.)
Please specify your answer.	(Free text response)
What was the primary method that you used in your most recent non-emergency euthanasia of a cat?	Pentobarbitone sodium<br>Anaesthetic inhalation<br>Potassium chloride<br>Thiopentone<br>Other (please specify)<br>(If you have used a combination of drugs, please select each of these.)
Please specify your answer.	(Free text response)
What was the route of administration of your chosen euthanasia drug?	Intravenous injection<br>Inhalation<br>Intracardiac injection<br>Intramuscular injection<br>Intraperitoneal injection<br>Oral administration<br>Subcutaneous injection<br>Other (please specify)

Please specify your answer.	(Free text response)
Was the euthanasia a house call or did it happen at the clinic?	House call<br>At the clinic<br>Other (please specify)
Please specify your answer.	(Free text response)
Was the owner present during the euthanasia?	Yes<br>No
How long do you schedule for a routine euthanasia?	10 minutes<br>20 minutes<br>30 minutes<br>Other (please specify)
Please specify your answer.	(Free text response)
Were you assisted during the euthanasia?	Yes<br>No
Who assisted?	Client<br>Veterinary nurse<br>Other (please specify)
Please specify your answer.	(Free text response)
What adjunctive measures did you take to minimise fear/anxiety/stress in the patient?	Away from other animals<br>Pheromones<br>Dim lighting<br>Longer appointment time<br>Soft bedding<br>Soft music playing<br>Treats<br>Cat only consult room<br>ISFM accreditation<br>Other (please specify)<br>None<br>(Please select all that apply)
Please specify your answer.	(Free text response)
Did you dispense medication to the owner for the patient prior to the appointment?	Barbiturates<br>Clonidine<br>Gabapentin<br>Opioids<br>Oral acepromazine<br>Trazodone<br>Other (please specify)<br>None<br>(If you have used a combination of drugs, please select each of these.)
Please specify your answer.	(Free text response)
Emergency Euthanasia	
For the last emergency euthanasia performed in a cat in the last 12 months, did you administer a premedication?	Yes<br>No<br>Have not performed
What was the reasoning behind using a premedication?	Chemical restraint<br>Clinic's protocols<br>Reduce stress to owner<br>Reduce stress to the patient<br>Taught to administer a premedication prior to euthanasia drugs<br>Other (please specify)<br>(Please select all that apply.)
Please specify your answer.	(Free text response)
What was the drug used for premedication prior to euthanasia under an emergency?	Acepromazine<br>Alfaxalone<br>Inhalation anaesthesia

	Ketamine<br>Medetomidine / Dexmedetomidine<br>Opioids (Methadone, buprenorphine, tramadol, butorphanol, pethidine)<br>Propofol<br>Tiletamine-zolazepam<br>Thiopentone<br>Xylazine<br>Other (please specify)<br>(If you have used a combination of drugs, please select each of these.)
Please specify your answer.	(Free text response)
What was the route of administration for the premedication that you used?	Inhalation<br>Intracardiac injection<br>Intravenous injection<br>Intramuscular injection<br>Intraperitoneal injection<br>Oral administration<br>Subcutaneous injection<br>Other (please specify)<br>(Please select all that apply.)
Please specify your answer.	(Free text response)
Was the emergency euthanasia a house call or did it happen at the clinic?	House call<br>At the clinic<br>Other (please specify)
Please specify your answer	(Free text response)
Was the owner present during the euthanasia?	Yes<br>No
Were you assisted during the euthanasia?	Yes<br>No
Who assisted?	Client<br>Veterinary nurse<br>Other (please specify)
Please specify your answer.	(Free text response)
What adjunctive measures did you take to minimise fear/anxiety/stress in the patient?	Away from other animals<br>Pheromones<br>Dim lighting<br>Longer appointment time<br>Soft bedding<br>Soft music playing<br>Treats<br>Cat only consult room<br>ISFM accreditation<br>Other (please specify)<br>None<br>(Please select all that apply.)
Please specify your answer.	(Free text response)
Is there anything else you wish to add about your approach to euthanising a cat?	(Free text response)
Demographics	
Gender:	Male<br>Female<br>Other
Number of years since graduation:	(Free text response) (Please enter a whole number)
Please select the most suitable option that best describes your primary workplace.	Animal Shelter practice/charity/NGO<br>Private companion animal practice<br>Private mixed practice<br>Research laboratory

	Veterinary teaching hospital<br>Other (please specify)
Please specify your answer.	(Free text response)
Please select the most suitable option that best describes the geographical location of your workplace.	Metropolitan (Major capital cities)<br>Regional (All of the towns, small cities and areas that lie beyond the major capital cities)<br>Rural (Open country and settlements fewer than 2,500 residents)<br>Remote (Places that are considerably out of the way and excluded from civilization)

Participant email address collection survey	
If you wish to receive the overall results of this study, please provide us with your email address and submit this survey.	(Free text response)

Table S2. Descriptive statistics of euthanasia practices of the most recent non-emergency euthanasia of a cat by Australian veterinarians (n = 615).

	Category	Number	Percentage (%)
Have you euthanised a cat in the last 12 months? (n = 615)	Yes<br>No	585<br>30	95.1<br>4.9
For the most recent, non-emergency euthanasia you performed in a cat, did you use premedication or sedation? (n = 585)	Yes<br>No	415<br>170	71.0<br>29.0
What was the reasoning behind using a premedication? (n = 415) (Participant could select multiple options)	Chemical restraint<br>Clinic's protocols<br>Reduce stress to owner<br>Reduce stress to patient<br>Taught to administer a premedication prior to euthanasia drugs<br>Other (please specify) (reduce operator stress n=6, minimise adverse effects n=5, analgesia n=3, combination of reasons n=3, no assistant n=2, aggression n=1, anaesthesia for intraorgan injection n=1, "CKD no veins" n=1, "Fear Free certification" n=1, "its what I always do, kinder" n=1, safety n=1, owner's request n=1).	242<br>54<br>327<br>388<br>32<br>26	58.3<br>13.0<br>78.6<br>93.3<br>7.7<br>6.3
What was the drug used for premedication or sedation prior to the euthanasia you performed most recently? (n = 415) (Participant could select multiple options)	Acepromazine<br>Alfaxalone<br>Inhalation anaesthesia<br>Ketamine<br>Medetomidine / Dexmedetomidine<br>Opioids (Methadone, buprenorphine, tramadol, butorphanol, pethidine)<br>Propofol<br>Tiletamine-zolazepam<br>Thiopentone<br>Xylazine<br>Other (please specify) (midazolam n=8, irrelevant n=2, gabapentin n=1, combined acepromazine and pentobarbitone n=1, combined tiletamine/zolazepam with pentobarbitone followed by tiletamine zolazepam with acepromazine n=1, pentobarbitone n=1, local anaesthetic (prilocaine) n=1.	120<br>61<br>2<br>31<br>61<br>118<br>20<br>205<br>10<br>9<br>15	28.9<br>14.7<br>0.5<br>7.5<br>14.7<br>28.4<br>4.8<br>49.4<br>2.4<br>2.2<br>3.6

What was the route of administration for the premedication that you used? (n = 415) (Participant could select multiple options)	Inhalation	2	0.5
	Intracardiac injection	1	0.2
	Intravenous injection	115	27.7
	Intramuscular injection	161	38.8
	Intraperitoneal injection	2	0.5
	Oral administration	4	0.95
	Subcutaneous injection	148	35.7
	Other (please specify)	0	0
What was the primary method that you used in your most recent non-emergency euthanasia of a cat? (n = 585) (Participant could select multiple options)	Pentobarbitone sodium	584	99.8
	Anaesthetic inhalation	2	0.3
	Potassium chloride	1	0.2
	Thiopentone	3	0.5
	Other (please specify) (firearm n=1)	0	0.9
What was the route of administration of your chosen euthanasia drug? (n = 585) (Participant could only select one option)	IV injection	536	91.6
	Inhalation	0	0
	Intracardiac injection	15	2.6
	Intramuscular injection	1	0.2
	Intraperitoneal injection	7	1.2
	Oral administration	0	0.0
	Subcutaneous injection	0	0.0
	Other (please specify) (intra-renal n= 23, hepatic n=1, nil drugs n=1, irrelevant n=1).	26	4.4
Was the euthanasia a house call or did it happen at the clinic? (n = 585) (Participant could only select one option)	House call	49	8.4
	At the clinic	531	90.8
	Other (please specify) (shelter or pound n=5)	5	0.85
Was the owner present during the euthanasia? (n = 585)	Yes	513	87.7
	No	72	12.3
How long do you schedule for a routine euthanasia? (n = 585) (Participant could only select one option)		22	3.8
	10 minutes	113	19.3
	20 minutes	345	59.0
	30 minutes	28	4.8
	40 minutes	23	3.9
	60 minutes	39	6.7
	Other (please specify) (10-30 mins n=1, 15 minutes n=4, ,30 minutes with nurse and 15 minutes with veterinarian n=1, 15-20 minutes n=1, 30-45 minutes n=1, 30-60 minutes n=1, 40-60 minutes n=3, 45 minutes n=8, 40-60 minutes n=1, 90 minutes n=1, daily time scheduled for euthanasia of shelter animals n=1, do not schedule appointments (walk in appointments or emergencies) n=9, minimum 30 minutes but owner can stay as long as they want n=1, unspecified n=4, aggressive feral cat dropped off n=1, irrelevant n=1.		
	Unlimited	15	2.6
Were you assisted during the euthanasia? (n = 585)	Yes	413	70.6
	No	172	29.4
Who assisted? (n = 413) (Participant could only select one option)	Client	3	0.7
	Veterinary nurse	405	97.8
	Other (please specify) (animal attendant or shelter staff n=3, veterinary student n=2).	5	1.2

What adjunctive measures did you take to minimise fear/anxiety/stress in the patient? (n = 585) (Participant could select multiple options)	Away from other animals	507	86.7
	Pheromones	207	35.4
	Dim lighting	128	22.0
	Longer appointment time	355	60.7
	Soft bedding	429	73.3
	Soft music playing	22	3.8
	Treats	81	13.8
	Cat only consult room	141	24.1
	ISFM accreditation	33	5.6
	Other (please specify) (private quiet room away from clinical activity n=5, allow owner to hold the cat n=5, gentle handling n=5, encourage owner to stay n=4, apply local anaesthetic prior to intravenous catheterization n=3, animal's own bedding n=2, aromatherapy or essential oils n=2, cover cat carrier n=2, candle in room n=2, house-call n=2, accommodate owner requests n=1, gentle speech n=1, calm approach n=1, minimise waiting time n=1, put up notice in reception n=1, place catheter away from owner n=1, place catheter with extension port n=1, pre-med cat where they are lying so not to disturb them n=1, sensible animal handling n=1, talk to owner to encourage a positive state of mind which may influence their cat n=1).	42	7.2
	None	26	4.4
Did you dispense medication to the owner for the patient prior to the appointment? (n = 585) (Participant could select multiple options)	Barbiturates	1	0.2
	Clonidine	0	0.0
	Gabapentin	51	8.7
	Opioids	2	0.3
	Oral acepromazine	1	0.2
	Trazodone	0	0.0
	Other (please specify)	0	0.0
	None	529	90.4

Table S3. Descriptive statistics of euthanasia practices of the most recent emergency euthanasia of a cat by Australian veterinarians (n = 585).

	**Category**	**Number**	**Percentage (%)**
For the last emergency euthanasia performed in a cat in the last 12 months, did you administer a premedication? (n = 585) (Participant could only select one option)	Yes	242	41.4
	No	220	37.6
	Have not performed	123	21.0
What was the reasoning behind using a premedication? (n = 242) (Participant could select multiple options)	Chemical restraint	139	57.4
	Clinic's protocols	24	9.9
	Reduce stress to owner	163	66.5
	Reduce stress to the patient	218	90.1
	Taught to administer a premedication prior to euthanasia drugs	11	4.5
	Other (please specify) (Analgesia n=14, reduce operator stress n=4, cat in respiratory distress n=3, safety n=2, to facilitate intravenous access in dehydrated kitten n=1).	24	9.9

What was the drug used for premedication prior to euthanasia under an emergency? (n = 242) (Participant could select multiple options)	Acepromazine	43	17.8
	Alfaxalone	45	18.6
	Inhalation anaesthesia	7	2.9
	Ketamine	21	8.7
	Medetomidine / Dexmedetomidine	35	14.4
	Opioids (Methadone, buprenorphine, tramadol, butorphanol, pethidine)	107	44.0
	Propofol	13	5.4
	Tiletamine-zolazepam	87	35.9
	Thiopentone	8	3.3
	Xylazine	5	2.1
	Other (please specify) (Midazolam n=1)	1	0.4
What was the route of administration for the premedication that you used? (n = 242) (Participant could select multiple options)	Inhalation	8	3.3
	Intracardiac injection	2	0.8
	Intravenous injection	92	38.0
	Intramuscular injection	107	44.2
	Intraperitoneal injection	2	0.8
	Oral administration	0	0.0
	Subcutaneous injection	45	18.6
	Other (please specify) (intra-renal n=2)	2	0.8
Was the emergency euthanasia a house call or did it happen at the clinic? (n = 242) (Participant could only select one option)	House call	13	5.4
	At the clinic	229	94.6
	Other (please specify)	0	0.0
Was the owner present during the euthanasia? (n = 242)	Yes	197	81.4
	No	45	18.6
Were you assisted during the euthanasia? (n = 242)	Yes	168	69.4
	No	74	30.6
Who assisted? (n = 168) (Participant could only select one option)	Client	5	3.0
	Veterinary nurse	162	96.4
	Other (please specify) (my daughter, n=1).	1	0.6
What adjunctive measures did you take to minimise fear/anxiety/stress in the patient? (n = 242) (Participant could select multiple options)	Away from other animals	206	85.2
	Pheromones	78	32.1
	Dim lighting	48	19.8
	Longer appointment time	116	47.7
	Soft bedding	165	67.9
	Soft music playing	7	2.9
	Treats	24	9.9
	Cat only consultation room	44	18.1
	ISFM accreditation	11	4.9
	Other (please specify) (aromatherapy or essential oils n=2, gentle handling n=2, owner present n=2, perform euthanasia quickly n=2, analgesia n=1, candle in the room n=1, apply local anaesthetic prior to intravenous catheterization n=1, gentle handling and conversation n=1, minimal handling with use of towels n=1, under general anaesthesia already n=1)	14	5.8
	None	17	7.0
